# Exploring volatility transmission in Capesize freight contracts: Insights from energy and commodity markets

**DOI:** 10.1371/journal.pone.0317487

**Published:** 2025-01-24

**Authors:** Jackson Jinhong Mi, Shek Ahmed, Yanhui Chen

**Affiliations:** 1 School of Economics & Management, Shanghai Maritime University, Shanghai, China; 2 Department of Mathematics, University of Barishal, Barisal, Bangladesh; Aalto University, FINLAND

## Abstract

Analyzing the interactions between spot and time charter freight is crucial for the maritime industry. While numerous studies have explored the relationship between average freight indices and spillover effects, a gap remains in understanding the deeper connections between inter-regional shipping routes and chartering contracts. This research investigates the role of Capesize freight dynamics in shaping the regional dry bulk freight market, with a focus on the influence of energy and commodity price fluctuations. Utilizing the TVP-VAR model, we identify distinct trends across various investment horizons. The analysis reveals that short-term spillovers dominate the system, with crude oil serving as a consistent shock transmitter within the time charter network. The China-Brazil route drives spillovers across all periods, while the Australia-China route transitions from absorbing short-term volatility to transmitting long-term shocks. Similarly, the Tubarão-Rotterdam and Bolivar-Rotterdam routes display comparable shifts, transmitting short-term spillovers but absorbing long-term volatility. These findings offer valuable insights for stakeholders seeking to manage risks amidst economic and geopolitical uncertainties.

## 1. Introduction

The integration of global shipping network into the global economy has been a significant trend, fostering diverse industries involved in intra-regional and foreign trade [1–4]. Maritime transportation has become the primary mode of global trade, facilitating over 90% of the total trade volume (UNCTAD, 2022). Unlike the oil transportation market, the dry bulk sector is characterized by intense competition and unpredictable fluctuations in freight rates, offering both opportunities and risks for market participants who transport more than one-third of the global shipping trade volume [5,6]. The advancing globalization and organizational structure of global industries have increased the influence of the shipping market and its cyclical fluctuations on the global economy. Therefore, it is essential to explore the economic intricacies of the shipping industry.

The Baltic Capesize Index (BCI) is an important indicator of freight revenue in the cargo market [7,8]. This market mainly involves the shipment of iron ore and coal, which accounts for 70–80% of iron ore and 27% of the total seaborne trade volume (UNCTAD, 2022). The changes in the volume of iron ore trade and fluctuating future settlement prices directly impact global commodity demand and the shipping market [7,9]. Since Chinese iron ore trade volume represents 70% of global imports, the demand for iron ore in China significantly influences the transportation market in the dry bulk industry and future iron ore price markets. Consequently, changes in the freight contract along the shipping route between China and its import partners can significantly impact the trade volume of dry bulk industry [10,11]. The major crude oil expense (50%) in shipping transportation within the shipping sector, where the Capesize sector holds a significant share in dry bulk shipping, is particularly vulnerable to fluctuations in oil prices [12]. This connection highlights the interdependence of the dry bulk freight with changes in iron ore and oil price markets.

There needs to be more research on the spillover linkages among dry bulk (BDI), crude oil, and iron-ore prices. Prior studies have primarily focused on mean spillover models such as Time-Varying Parameter Vector Autoregressive (TVP-VAR) and the multi-dimensional GARCH model to illustrate the asymmetric impact of spillover indicators. However, these studies still need to address the time-frequency domain spillover interaction among networks. For instance, Chen et al. [9] utilized the BEKK-GARCH-X approach to examine time-varying inter-relationships among dry bulk freight (BDI), crude oil, and the iron-ore market. They identified dynamic lead-lag relationships in the system, each variable serving as an exogenous variable for different sub-samples. Nevertheless, their findings do not offer insight into the direction of spillover interconnectedness across time and frequency shocks. Moreover, using average spot freight for dry bulk cannot capture the individual characteristics of the transportation market across diverse chartering freight.

The Time-Varying Parameter Vector Autoregressive (TVP-VAR) model, designed for time-varying dynamics, excels in capturing evolving relationships and volatility spillovers in non-stationary environments. Given that the freight and commodity markets experience structural changes due to economic cycles, policy shifts, and fluctuating supply-demand conditions, TVP-VAR is ideal for modeling such dynamic interactions over time [13–15]. The static result can evaluate the average spillover outcome over a specific period. offers several advantages over the mean-based estimation analysis. While Bayesian VAR allows for the incorporation of prior knowledge and is effective with small sample sizes, its static parameter approach is less suited for capturing the evolving dynamics of volatility transmission. Given the complexity of freight and commodity markets, which are subject to significant and abrupt shifts, a time-invariant model may oversimplify the interdependencies [16–18].

Dynamic Conditional Correlation (DCC) is excellent for analyzing time-varying correlations, especially in financial time-series data. However, DCC primarily focuses on estimating conditional correlations rather than the direct impact of shocks from one variable on another over time, which is central to my research on volatility transmission [19–23]. While Generalized Impulse Response Function (GIRF) is valuable for examining the response of systems to shocks, it assumes time-invariant parameters [24–27]. In contrast, TVP-VAR provides the flexibility to assess how these responses evolve over time, making it more applicable in markets characterized by volatility clusters and regime shifts. Table 1 provides a detailed comparison of the characteristics of the mentioned models, offering readers a clearer understanding of their distinct features and applications.

**Table 1:** Model comparison.

Model	Characteristics	References
TVP-VAR	Non-stationary, Time-varying high-frequency evolving systems	Antonakakis et al. [13], Riaz et al. [14], Chatziantoniou et al. [15], Jiang et al. [28]
Bayesian VAR	Small sample sizes, Prior uncertainty	Assaf et al. [16], Apergis & Apergis [17], Simionescu et al. [18], Carriero et al. [29]
Dynamic Conditional Correlation (DCC)	High frequency data, High volatility correlation analysis, no direction analyze	Tsuji [19], Hung [20], Li & Yip [21], Yadav et al. [22], Ben et al. [23]
Generalized Impulse Response Function (GIRF)	Structural macroeconomic shocks in nonlinear systems	Wang & Dunne [24], Bisping & Patron [25], Balcilar [26], Zhang & Baek [27]

In summary, TVP-VAR is selected because it aligns well with the study’s goal of capturing the dynamic and time-varying nature of volatility transmission across interconnected markets. This choice allows for a nuanced analysis that better reflects real-world fluctuations in the Capesize freight, energy, and commodity sectors, ultimately providing more robust insights into their interdependencies. This model not only evaluates the spillover index of the overall system but also examines the transmission flow of volatility effect to individual variable. As the spillover effect varies by window size in the rolling window VAR technique, this may lead to biased measurements of spillover estimations.

The current analysis fails to capture variations in the sizes and directions of spillover impacts across diverse situations and does not account for spillover effects in the case of heterogeneous price returns [13]. In the TVP-VAR spillover connection model, Antonakakis et al. [13] demonstrated that the network connection model has greater handling power in using time series data, more robust forecasting, and is faster and more flexible in reaction to financial-economic indicators compared to the spillover overflow model. Various studies [30–33] on spillover connection have shown that the spillover impact of economic variables fluctuates over time during financial crises and has a substantial influence on the GDP.

This article uses time and frequency connectedness model introduced by Chatziantoniou et al. [15] which can capture the dynamic connectedness over the network and pairwise connection among multi-freight charter periods [34]. Therefore, employing the Time-Varying Parameter Vector Autoregressive model incorporating Diebold & Yilmaz [35,36] spillover index method (TVP-VAR-DY) and Time-Varying Parameter Vector Autoregressive model based on Baruník and Křehík [37] frequency connectedness approach (TVP-VAR-BK) to investigate time and frequency domain spillover connectedness among spot voyage/time charter, energy, and commodity returns is essential. These findings offer vital information to diversify portfolios during financial crises and other economic shocks, enabling policymakers to manage the market more effectively across diverse frequencies. Therefore, this model is more effective than other techniques in analyzing the spillover connectedness within spot voyage and time charter freight, including energy and commodity futures markets.

This article delves into the impact of the oil crisis 2016, the US-China trade war in 2018, and COVID-19 on the interconnectedness of energy, commodities, and diversified freight markets. This study employs time and frequency domain TVP-VAR technique introduced by Palaios et al. [33] and Diebold & Yilmaz [35,36]. Moreover, the study emphasizes the importance of considering the time-frequency domain investment component when studying connectivity due to regional diversity in spot voyage/time charter freight. Applying BK spillover overflow model, which correctly describes shocks of economic variables in varying strengths by diverse frequencies, an improvement over the TVP-VAR-DY model.

This study seeks to fill an existing research gap by examining these dynamics and exploring the connections during specific timeframes. Furthermore, the study broadens its focus beyond freight markets to include various regional transportation sectors that have seen significant price fluctuations during major economic and geopolitical disruptions. Our analysis indicates robust short-run spillover connections among chartering freight, energy, and commodity prices, especially during economic downturns. Within the time charter framework, crude oil prices serve as consistent transmitters of short- and long-term shocks however altering their spillover direction from receiver to transmitter over time along voyage charter route. The China-Brazil route, encompassing both voyage and time charter routes, emerges as a primary conduit for spillover transmission across short- and long-term horizons. Conversely, the Australia-China route absorbs short-term volatility but transitions to transmitting shocks over the long term. Additionally, the Tubarao-Rotterdam and Bolivar-Rotterdam routes transmit short-term volatility but receive significant volatility in the long term. These insights provide policymakers and shipping industry stakeholders with valuable guidance for developing risk mitigation strategies amid economic and geopolitical instability.

This paper is divided into the following sections: section 2 reviews relevant literature; section 3 outlines the method; section 4 discusses description of the data; section 5 gives the empirical findings and addresses robustness where section 6 concludes findings and policy implications.

## 2. Review of literature

The dry bulk market saw a significant revival and subsequent surge from 2014 to September 2023, followed by a steep decline because of the oil, covid-19 and geopolitical crisis [38–41]. Fluctuating freight rates have a direct impact on market participants, such as shipowners, charterers, and investors, affecting their expenses and profits. It is crucial to understand how information moves within the Capesize market, which is a major trading route for the cargo industry, in order to manage operating costs and deal with volatility in return shocks. This understanding requires analyzing how changes in energy and commodity markets and external factors that influence the market affect spot voyage and time charter contracts. Stakeholders depend on this knowledge to make informed decisions and navigate the intricacies of the freight market in both the short and long term.

### 2.1 Interlinkage between crude oil and dry bulk market

The BDI is a key measure of global freight indices that reflects commodity trade volumes and closely aligns with crude oil market changes. Crude oil prices were immediately reflected in the BDI using worldwide shipping and transportation expenses. Conversely, fluctuations in global market variables such as the global economic cycle, business cycle, and significant crises would simultaneously impact the cargo freight and global energy price, initiating a synchronized trend of volatility in both markets. The dry bulk and crude oil price exhibited a notable co-mobility, with the short-term shocks being more pronounced than the long-term ones.

There are intriguing studies that primarily explore the pricing mechanisms of crude oil future and how it interacts with other dry bulk shipping markets amid considerable economic and geopolitical uncertainties [9,14,42–44]. For example, Diebold & Yilmaz [35] built numerous scenarios of the impact of high and low BDI and oil prices and reached comparable findings. Shi et al. [45] developed a stochastic Vector Autoregressive (VAR) model and demonstrated that the tanker market saw substantial effects from shocks in crude oil supply. Lee et al. [2,46] reaffirm that oil prices contributed to persistent higher volatility spillovers in seaborne transportation. In accordance with this, Shi et al. [47] and Riaz et al. [14] use the TVP-VAR DY model and demonstrate that energy fluctuations have a more significant spillover effect on the tanker freight compared to the dry bulk market. However, their research reveals smaller vessels exhibit more volatility than large vessels. Sun et al. [1] utilize the dynamic conditional correlation GARCH technique to analyze volatility and transmission impact among energy, bunker fuel and freight markets. Their results demonstrate asymmetric patterns of returns and volatility of energy futures as the primary volatility spillovers.

According to Zhang [48], shifting crude prices significantly affects bunker prices and the shipping freight market. They demonstrate that rising oil prices lessens the inter-dependency between derivative markets. Sun et al. [49] comprehensively analyzes the volatility interaction between energy Futures and crude oil and clean tankers using monthly data and reveals a heightened association between oil price shocks and the tanker freight market. Kobougia & Kyrkilis [50] employ OLS techniques to analyze how Chinese dry bulk seaborne imports, production level, bulker fleet, and port throughput affect dry bulk freight. In their recent study, Angelopoulos et al. [51] employ time varying factor framework and present that oil market functions as the primary factor influencing commodity and shipping markets. Lin et al. [52] utilizes the GARCH-MIDAS approach and demonstrates that dry bulk, tanker freight and commodity markets affect the crude oil price market returns.

The spillover connectivity was more obvious during the US-China trade war and persisted during the Russia-Ukraine conflict. Cheng et al. [38] and Sun et al. [53] use optimal hedging techniques and found significant volatility transmission between dry bulk and crude oil futures. Some researchers utilize the time and frequency domain TVP-VAR model and demonstrate that short-term crude oil shocks dominate maritime freight market volatility [36,54]. Few studies [55–57] reveal that uncertainty indicators such as geopolitical risk, economic policy, and climate policy have a short-term impact on BDI and crude oil price.

### 2.2 Interlinkage between iron-ore and dry bulk market

The extensive use of sea transportation in commodity shipping creates an inevitable link between commodity prices and maritime markets. Variations in commodity prices impact global economic demand and the freight market. Previous studies have revealed time-variable connections between freight chartering contract and commodity futures. There exists a significant link between the two as studies by Kavussanos et al. [58]. Their empirical study provided evidence of strong cointegration and show that agricultural commodities highly influence dry bulk freight rate. Michail & Melas [59] studied the spillover connection by examining how agricultural commodity prices influence the time charter market in the Capesize industry. Using QVAR time-frequency connectedness, Adewuyi et al. [60] showed a heightened link between the dry bulk industry and the commodities market. In the short term, shipping markets play a dominant role as both the transmitters and receivers of the most significant shocks to the entire market system; however, the long-term connection demonstrates limited connectedness.

The time variable quantile analysis reveals that the connection was particularly strong before, during, and after the COVID-19 crisis. Tsioumas & Papadimitriou [61] extensively studied the relationship between metals and the dry bulk voyage freight and concluded that iron ore and coal have a two-way connection with the Baltic Dry index compared to agricultural commodities. Gu et al. [7] also analyzed the association between BDI and iron ore spot price using the vector error correction model, showing a strong link between the freight rate and commodity futures. A study by Khan et al. [44] found that shocks from crude oil and agricultural commodity markets affect shipping freight and metal commodities, especially during challenging market conditions. Tiwari et al. [62] utilized the mean-variance causality technique to demonstrate that dry bulk freight is a significant transmitter of volatility in most commodities indexes.

By applying the BEKK-GARCH model, Lin et al. [52] revealed that dry bulk is a short-term volatility transmitter in both stock and commodities markets. Furthermore, the authors utilized the GARCH-BEKK method to demonstrate that dry bulk spot freight acts as a short-term volatility transmitter during global crises in commodity and financial markets. Acik & Baser [63] examined volatility spillover among commodity prices and Capesize voyage freight using the Granger causality technique and identified iron ore and coal prices as the sole causes of volatility in freight rates. They also employed the VAR approach to illustrate that positive shocks in iron ore prices lead to negative shocks, while adverse shocks result in negative and positive shocks in the Capesize spot voyage freight rate.

## 3. Methods

### 3.1 Time domain TVP-VAR method

We measure the volatility spillovers of the freight market across different routes in terms of spot voyage and time charter freight using the TVP-VAR model presented by Antonakakis et al. [13]. We have utilized a combination of the volatility spillover index approach developed by Diebold & Yilmaz [35,64] with a time-varying element to achieve our goal. The original approach put forth by Diebold and Yilmaz had some limitations in terms of methodology, such as its reliance on variable orderings due to its basis on Cholesky factor orthogonalization in a VAR estimation framework. While this statistical technique is effective in generating constant directional spillovers of each variable in the model assuming stability throughout the entire period, it cannot estimate the time-varying changes of the parameter.

This study handles dynamic changes in VAR equation parameters over time by adapting the TVP-VAR DY approach. By eliminating the need for a predetermined window width, the risk of sample loss is significantly reduced. As a result, this approach effectively mitigates the limitations of the volatility spillover index method introduced by Diebold & Yilmaz [33,34,64]. The TVP-VAR(p) model can be expressed in the following manner:

$$x_{t}=Q_{t}y_{t-1}+\emptyset_{t}\;\emptyset_{t}|\delta_{t-1}∼N(0,\Delta_{t})$$ (1)

$$vec(Q_{t})=vec(Q_{t-1})+\varepsilon_{t}\;\varepsilon_{t}|\delta_{t-1}∼N(0,\nabla_{t})$$ (2)

$$with\;y_{t-1}=\left(\begin{array}{c} x_{t-1}\\ x_{t-2}\\ x_{t-p} \end{array}\right)\;{Q\prime }_{t}=\left(\begin{array}{c} Q_{1t}\\ Q_{2t}\\ Q_{pt} \end{array}\right)$$ (3)

where *δ*_*t*−1_ indicate all market information available until *t*−1 with *x*_*t*_,*y*_*t*−1_,*Q*_*t*_,*Q*_*it*_ signifies matrices of order *m*×1,*mp*×1,*m*×*mp* and *m*×*m* respectively. The error vectors ∅_*t*_,*ε*_*t*_ are matrices of order *m*×1,*m*^2^*p*×1 whereas the time-varying variance-covariance matrices Δ_*t*_ and ∇_*t*_ represent order *m*×*m* and *m*^2^*p*×*m*^2^*p* respectively. Moreover, *vec*(*Q*_*t*_) is the vectorization of *Q*_*t*_ of order *m*^2^*p*×1. Based on Eqs (3) and (4), we decompose the H-step-ahead prediction error variance ($\vartheta_{ij}^{k}(H)$) and evaluate the spillover effect of freight rate *X*_*j*_ due to changes in market rate *X*_*i*_ for *i*≠*j*. Hence, the H-step ahead error variance $\vartheta_{ij}^{k}(H)$ is calculated as

$$\vartheta_{ij}^{k}(H)=\frac{\theta_{jj}^{-1}\sum_{h=0}^{H}{\left({z\prime }_{i}Q_{h}\delta z_{j}\right)}^{2}}{\sum_{h=0}^{H-1}z_{j}^{H-1}({z\prime }_{j}Q_{h}\delta {Q\prime }_{h}z_{i})}$$ (4)

Among them, *δ* is the variance function of the error term ε. The value of the H-dimensional selection vector (*z*_*i*_) in ith position is 1 and the rest of the term is 0. To achieve a sum of one for each array within the spillover matrix, it is necessary to standardize the variance decomposition matrix for each shock applied to non-orthogonalized variables. This standardization process involves normalizing each entry of the matrix by the sum of its respective rows, ensuring that the variance decomposition of each row totals one:

$${\tilde{\upsilon }}_{ij}^{k}(H)=\frac{\vartheta_{ij}^{k}(H)}{\sum_{j=1}^{N}\vartheta_{ij}^{k}(H)}$$ (5)

where $\sum_{j=1}^{N}{\tilde{\upsilon }}_{ij}^{k}(H)=1\;and\;\sum_{i,j=1}^{N}{\tilde{\upsilon }}_{ij}^{k}(H)=N$.

The directional volatility spillover index “FROM” and “TO” received and emits volatility to other markets is estimated as:

$${FROM}_{i,From}(H)=\frac{\sum_{j=1,i\neq j}^{N}{\tilde{\upsilon }}_{ij}^{k}(H)}{\sum_{i,j=1}^{N}{\tilde{\upsilon }}_{ij}^{k}(H)}\times 100,{TO}_{i,To}(H)=\frac{\sum_{j=1,i\neq j}^{N}{\tilde{\upsilon }}_{ji}^{k}(H)}{\sum_{i,j=1}^{N}{\tilde{\upsilon }}_{ji}^{k}(H)}\times 100$$ (6)

Finally, the net volatility spillover index (*NVSI*) is calculated using Eq (7). The positive value of indicates net volatility transmitter of volatility shocks and the negative value represents the net receiver of volatility shocks. The net volatility spillover index from and to the *i*th market is estimated as

$${Net}_{i}={TO}_{i,To}(H)-{FROM}_{i,From}(H)$$ (7)

The total volatility spillover index which measures the overall volatility spillover correlations of all variables in the shipping market is calculated as

$$TCI(H)=\frac{\sum_{i,j=1,i\neq j}^{N}{\tilde{\upsilon }}_{ij}^{k}(H)}{\sum_{i,j=1}^{N}{\tilde{\upsilon }}_{ij}^{k}(H)}\times 100=\frac{\sum_{i,j=1,i\neq j}^{N}{\tilde{\upsilon }}_{ij}^{k}(H)}{N}\times 100$$ (8)

To account for changes in volatility spillovers caused by fluctuations in the economic environment, this study uses a time-varying spillover method (TVP-VAR-DY) developed by Antonakakis et al. [13]. We analyze volatility spillovers with a rolling window size of 200 and a 20-week forecast horizon.

### 3.2 Frequency domain TVP-VAR method

The frequency connectedness models the time domain connectedness on several short-, medium, and long-term frequencies. The multi-frequency spillover analysis introduced by Baruník and Kˇrehlík [37] reflects a more precise integration compared to the time domain spillover technique introduced by Diebold & Yilmaz [35,36]. The segmented spillover connectedness analysis gives better accuracy in understanding the impact of short-, medium, and long-term shocks of individual indicators on total connectedness. Considering the limitation of the DY and BK model on modeling structure, Chatziantoniou et al. [15] proposed a generalized frequency connectedness model using the forecast error variance decomposition (FEVD) decomposition method on freight market volatility returns. The Fourier transformation of the TVP-VMA(∞) for the frequency response $\varphi (e^{-i\omega })=\sum_{h=0}^{\infty }e^{-i\omega h}$, is defined by

$$S_{x}(\omega )=\sum_{h=-\infty }^{\infty }E(y{y^{\prime }}_{t-h}{)e}^{-i\omega }h=\varphi_{t}(e^{-i\omega h})\sum_{t}{\varphi \prime }_{t}(e^{+i\omega h})$$ (9)

where *i* is a imaginary number equivalent to $\sqrt{-1}$, and ω denotes the set of frequency interval and *y*_*t*_ denotes spectral density. The normalized form of forecast error variance decomposition (FEVD) decomposition matrix defined as

$$\upsilon_{ij}^{g}(\omega )=\frac{{{(\Sigma }_{t})}_{ij}^{-1}{\left|\sum_{h=0}^{\infty }{{{(\varphi }_{t}(e}^{-i\omega h})\Sigma_{t})}_{ijt}\right|}^{2}}{\sum_{h=0}^{\infty }{{{(\varphi }_{t}(e}^{-i\omega h})\Sigma_{t}\varphi_{t}(e^{i\omega h}))}_{ii}}$$ (10)

$${\tilde{\upsilon }}_{ij}^{g}(\omega )=\frac{\theta_{ijt(\omega )}}{\sum_{k=1}^{N}\theta_{ijt(\omega )}}$$ (11)

The portion of spillover shocks for the changes of *j* th variable attributed on the *i* th variable denoted by ${\tilde{\theta }}_{ijt}(\omega )$. The whole spectrum frequencies can be segmented within a specific range

$$d=(x,y):x,y\in (-\pi ,\pi ),x<y\;and\;{\tilde{\upsilon }}_{ij}^{g}(d)=\int_{x}^{y}{\tilde{\upsilon }}_{ij}^{g}(\omega )d\omega$$ (12)

The whole spillover connectedness metrics can be defined as follows.


$${TO}_{it}(d)=\sum_{i=1,i\neq j}^{N}{\tilde{\upsilon }}_{jit}^{g}(d)$$ (13)

$${FROM}_{it}(d)=\sum_{i=1,i\neq j}^{N}{\tilde{\upsilon }}_{jit}^{g}(d)$$ (14)

$${Net}_{it}(d)={TO}_{it}(d)-{FROM}_{it}(d)$$ (15)

$${TCI}_{t}(d)=\frac{\sum_{i=1,i\neq j}^{N}{\tilde{\upsilon }}_{jit}^{g}(d)}{\sum_{i,j=1}^{N}{\tilde{\upsilon }}_{jit}^{g}(d)}$$ (16)

$${NPSC}_{ij}^{g}(d)=({\tilde{\upsilon }}_{jit}^{g}(d)-{\tilde{\upsilon }}_{ijt}^{g}(d))\times 100$$ (17)

The above spillover connectedness don’t reflect the overall connectedness. The indices of total, directional and net spillover connectedness for each band *d* are weighted with the whole system by the equation $\Gamma (d)=\frac{\sum_{i,j=1}^{N}{\tilde{\theta }}_{ijt(d)}}{N}$ which helps to measure better approximation of segmented shocks in diverse interval.


$${\tilde{T}O}_{it}=\Gamma (d).{TO}_{it}(d)$$ (18)

$${\tilde{F}ROM}_{it}=\Gamma (d).{FROM}_{it}(d)$$ (19)

$${\tilde{N}et}_{it}=\Gamma (d).{Net}_{it}(d)$$ (20)

$${\tilde{T}CI}_{t}=\Gamma (d).{TCI}_{t}(d)$$ (21)

$${\tilde{N}PSC}_{t}=\Gamma (d).{NPSC}_{t}(d)$$ (22)

This study set the short-term frequency to be 1–28 days and long-term frequency to be 28 days and above. This segmentation matches the practical application of shipping freight contracts in the shipping industry. The single voyage spot freight market and spot round voyage time charter freight in bulk transportation is considered due to the higher fluctuation of freight rates in the exporting and importing countries.

## 4. Variable description and descriptive statistics

This section provides a detailed description of the variables used in the analysis and their respective roles in understanding spillover dynamics within the shipping freight market. It introduces the data sources and outlines the key features of the variables, including spot and time charter freight indices, energy prices, and commodity markets. Additionally, the section presents descriptive statistics to summarize the central tendencies, variability, and distributional properties of the data. These statistics offer preliminary insights into the characteristics of the variables and their potential relationships, serving as a foundation for subsequent spillover analysis.

### 4.1 Variable description

**Fig 1:**
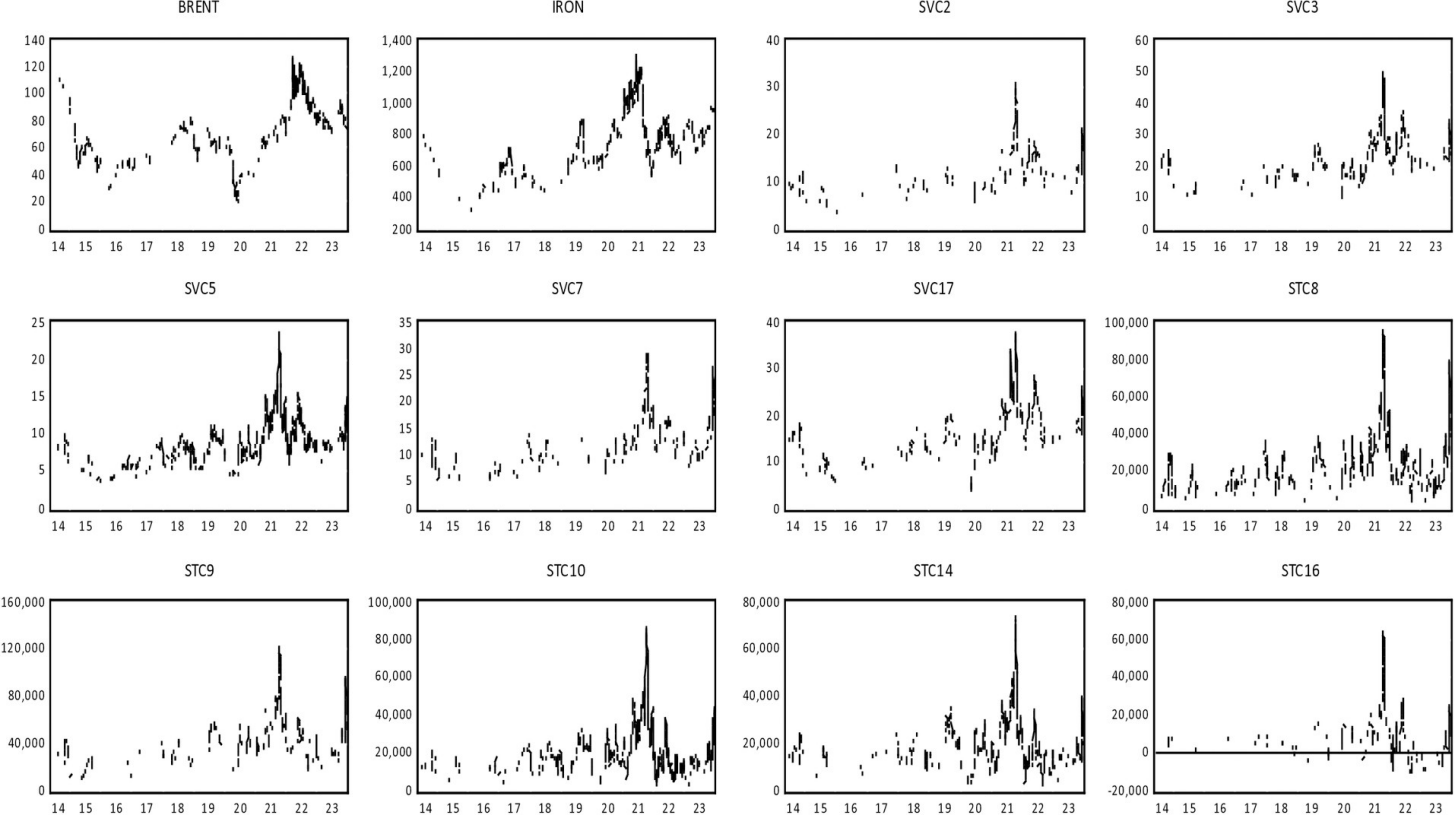
Time series behavior among freight contract, Brent crude oil and iron ore future market. Note: All time series are drawn from original series. SV represents the spot voyage freight market and ST represents spot time charter freight market. BRENT, IRON, SV2, SV3, SV5, SV7, SV17, SV8, ST9, ST10, ST14 and ST16 have individual market definition as illustrated in Table 1.

Our dataset consists of time-series data on daily spot voyages, spot time charters, and the closing prices of crude oil and iron ore futures. The Brent 1-month futures contract, an essential benchmark in global crude oil trading, is among the most actively traded energy futures. We have also included the iron ore 1-month futures contract, known for its high volatility in commodity markets, mainly cargo trading. To distinguish between spot voyage and time charter freight rates, we have focused on the shipping routes that exhibit the most significant fluctuations. The data details are presented in Table 2, covering the period from June 23, 2014, to December 21, 2023, with a total of 2,376 observations. Fig 1 shows the time series trajectory of the original series for our spot voyage and time charter freight, as well as future crude oil and iron-ore contract prices. All series exhibit substantial fluctuations, particularly during high economic and geopolitical uncertainty periods.

**Table 2:** Descriptive and correlation statistics.

Variable	BRENT	IRON	SVC2	SVC3	SVC5	SVC7	SVC17	STC8	STC9	STC10	STC14	STC16
Mean	-0.005	0.003	0.008	0.002	0.006	0.008	0.004	0.018	0.006	0.013	0.007	0.004
Median	0.047	0.032	-0.099	-0.076	-0.085	-0.117	-0.059	-0.255	-0.163	-0.143	-0.131	-0.055
Maximum	8.285	3.287	17.992	7.922	9.820	8.362	16.542	29.501	12.221	33.481	27.561	292.161
Minimum	-12.149	-7.028	-7.636	-5.099	-10.434	-7.636	-21.385	-36.972	-9.672	-22.932	-17.001	-214.611
Std.Dev	1.134	0.909	1.407	1.237	1.806	1.362	1.395	3.857	1.906	4.308	3.046	8.382
Skewness	-0.911	-1.176	1.469	0.692	0.191	0.762	-0.209	0.118	0.968	0.479	1.032	10.551
Kurtosis	18.112	10.027	18.750	6.693	5.346	7.741	46.302	15.959	8.359	9.578	12.861	810.196
JB test	22940.85**	5437.53**	25414.26**	1540.07**	559.62**	2455.84**	185654.7**	16632.66**	3215.39**	4375.80**	10049.36**	64549.1**
UR test	-13.06**	-12.61**	-11.91**	-12.73**	-12.77**	-11.87**	-12.45**	-12.51**	-12.43**	-12.16**	-12.01**	-12.84**
Q (30)	57.31**	58.70**	252.48**	160.86**	95.80**	202.81**	183.94**	224.04**	172.48**	99.895**	188.93**	2.1691
nobs	2376	2376	2376	2376	2376	2376	2376	2376	2376	2376	2376	2376
Pearson correlation coefficient												
BRENT	1.000											
IRON	0.082	1.000										
SVC2	0.004	0.002	1.000									
SVC3	-0.004	0.014	0.739	1.000								
SVC5	-0.007	0.021	0.571	0.651	1.000							
SVC7	0.007	0.033	0.781	0.675	0.521	1.000						
SVC17	-0.027	-0.003	0.621	0.728	0.550	0.577	1.000					
STC8	-0.015	0.034	0.680	0.590	0.447	0.854	0.496	1.000				
STC9	-0.025	0.020	0.732	0.727	0.529	0.794	0.605	0.763	1.000			
STC10	-0.016	0.031	0.553	0.625	0.874	0.512	0.526	0.509	0.547	1.000		
STC14	-0.010	0.025	0.679	0.844	0.669	0.635	0.649	0.661	0.702	0.782	1.000	
STC16	-0.007	0.016	0.227	0.192	0.171	0.182	0.156	0.303	0.219	0.231	0.352	1.000

**Notes:** (1) *, ** indicate the significant levels of 1% and 5% respectively. (2) All the time series data are derived from Clarkson and Wind data base (3) Since some freight index contain negative values, this paper uses simple returns instead of the logarithmic change.

### 4.2 Preliminary data analysis

Table 3 presents the descriptive and correlation statistics of the return series encompassing spot voyage, spot time charter and two future crude oil and iron-ore markets. Notably, the daily average returns for all freight markets are statistically insignificant, except for oil returns, which exhibit a negative mean. The standard deviation values in the table reflect the volatility within each series, with some, like STC8 (3.86), STC10 (4.31) and STC16 (8.38), showing much higher dispersion compared to others. This indicates that these variables experience greater fluctuations around their mean, suggesting a higher level of unpredictability or risk in these series. Finally, Brent crude oil future exhibits moderate level volatility and a dominant level of liquidity when compared to other markets, as supported by Wang et al.’s [65] research. The crude oil market stands out as a dominant market with an impressive level of liquidity compared to other markets. The study emphasizes the risks posed by round voyage freight markets and future crude oil and iron ore prices, which experienced lower average returns and higher volatility during the sample period. The kurtosis values (Table 3) highlight extreme peakedness and heavy tails for most variables, with especially high kurtosis in SVC17 (46.30) and STC16 (810.20), indicating the presence of frequent and significant outliers. Such high kurtosis suggests that these variables exhibit sharp deviations from a normal distribution, with data points clustered around the mean and in the tails, leading to a higher likelihood of extreme values. The skewness values indicate that several variables have asymmetrical distributions, with both positive and negative skewness. For example, STC10 has a high positive skewness (10.55), suggesting a distribution with a long right tail and frequent large positive values, whereas BRENT has a negative skewness (-0.91), indicating a leftward skew with more frequent lower values. The Pearson correlations indicate varying degrees of linear relationships among variables, with some pairs, like SVC3 and SVC2 (0.7397) and STC10 and SVC5 (0.8740), showing strong positive correlations. This suggests that these variables tend to move together, likely due to shared underlying economic or market influences. The JB test of Jarque and Bera [66] implies that no series is normally distributed, which is compatible with the statistical findings of skewness and kurtosis. The Q(30) findings of the Ljung-Box test [67] reveal evidence of strong serial correlation and heteroskedasticity in all series. The UR tests of Dickey & Fuller [68] revealed stationarity in all series and found ARCH/GARCH errors in some of the variables, based on the work of Dickey & Fuller [68]. Thus, the forecast error variance decomposition (FEVD) based spillover index approach (TVP-VAR-DY) is suited for these fat-tailed and volatility clustering time series.

**Table 3:** Description of variables.

Variable	Description	Unit
BRENT	Brent Oil Futures Settlement Price (Continuous): Brent Oil	US dollar/barrel
IRON	Futures Closing Price (Active Contract): Iron Ore	Yuan/Ton
SVC2	BCI C2: Tubarao/Rotterdam 160,000 long tons	US dollar/Ton
SVC3	BCI C3: Tubarao/Qingdao, 160,000 or 170,000 mt	US dollar/Ton
SVC5	BCI C5: W. Australia/Qingdao, 160,000 mt	US dollar/Ton
SVC7	BCI C7: Bolivar/Rotterdam 150,000 mt	US dollar/Ton
SVC17	BCI C17: Saldanha Bay-Qingdao, 170,000mt	US dollar/Ton
STC8	BCI C8_14: 180,000mt, Gibraltar/Hamburg transatlantic voyage	US dollar/Day
STC9	BCI C9_14: 180,000mt, Continent/Mediterranean trip China-Japan	US dollar/Day
STC10	BCI C10_14: 180,000mt, China-Japan transpacific voyage	US dollar/Day
STC14	BCI C14: China-Brazil round voyage, 180,000mt	US dollar/Day
STC16	BCI C16: Revised backhaul	US dollar/Day

## 5. Empirical findings

We employ the TVP-VAR model to analyze both static and dynamic spillover connectedness, utilizing a rolling window size of 200, an optimal lag length determined based on the Akaike Information Criterion (AIC) and forecast horizons are set at 20 steps ahead. The analysis of static spillovers is presented in the first section, while the discussion of dynamic spillovers and robustness is addressed in the third and fourth sections, respectively.

### 5.1. Average time and frequency domain spillover connectedness

The results mentioned in this section are generated using the average frequency connectedness method of the TVP-VAR DY and BK approach provided by Barunik and Krehlik [37]. This technique successfully captures dynamic linkages across multiple market frequencies, distinguishing between short-term (1–28 days) and long-term (beyond 28 days) effects, as detailed in Tables 3 and 4. The horizontal axis represents the time dimension, showing how the spillover effects evolve over time. The vertical axis represents the intensity of the spillover effect in terms of net pairwise directional connectedness. Higher values indicate stronger spillover effects from one variable to another. The (*i*,*j*)th item represents the expected contribution to prediction error variance i resulting from market innovation j. The spillovers contributed to other variables ’TO’ are computed using the off-diagonal column sum. In contrast, spillovers received from other variable ’FROM’ are estimated using the row sum, eliminating diagonal spillovers. The net spillover "Net" is produced by subtracting ’FROM’ from ’TO,’ and the total spillover connectedness index (TCI) is calculated by dividing off-diagonal spillovers by total spillovers, including diagonal spillovers.

**Table 4:** Time-frequency average spillover connectedness along spot time charter route.

	BRENT	IRON	STC8	STC9	STC10	STC14	STC16	FROM
Total spillover connectedness based on TVP-VAR-DY								
BRENT	92.35	2.16	1.18	1.25	1.00	1.12	0.95	7.65
IRON	4.58	87.16	1.63	2.02	1.61	2.21	0.79	12.84
STC8	1.04	0.98	43.12	23.95	11.25	19.16	0.5	56.88
STC9	0.93	0.82	21.55	40.88	13.02	21.87	0.94	59.12
STC10	0.57	0.74	11.41	13.91	43.44	29.14	0.78	56.56
STC14	0.66	0.77	15.36	19.14	24.05	39.43	0.6	60.57
STC16	0.75	0.41	0.99	2.1	1.12	1.29	93.33	6.67
TO	8.53	5.88	52.11	62.36	52.04	74.8	4.56	260.29
Net	0.88	-6.96	-4.76	3.24	-4.52	14.23	-2.11	TCI = 37.18
NPDC	1	0	4	5	3	6	2	
Short-term (1–28 Days) spillover connectedness based on TVP-VAR-DY-BK								
BRENT	88.13	2.07	1.12	1.14	0.95	1.03	0.88	7.18
IRON	4.3	81.24	1.46	1.8	1.5	2.03	0.71	11.8
STC8	0.86	0.83	35.97	19.2	9.47	15.62	0.43	46.41
STC9	0.78	0.67	17.41	33.78	11	17.93	0.83	48.62
STC10	0.5	0.64	9.69	11.92	39.12	25.76	0.75	49.26
STC14	0.55	0.64	12.66	15.89	20.91	33.89	0.55	51.19
STC16	0.72	0.39	0.89	1.91	1.01	1.16	88.45	6.08
TO	7.71	5.23	43.23	51.86	44.83	63.53	4.15	220.54
Net	0.53	-6.57	-3.18	3.24	-4.43	12.34	-1.92	TCI = 31.51
NPDC	1	0	4	5	3	6	2	
Long-term (28 Days and above) spillover connectedness based on TVP-VAR-DY-BK								
BRENT	4.23	0.09	0.06	0.1	0.05	0.09	0.07	0.47
IRON	0.29	5.92	0.17	0.22	0.11	0.18	0.08	1.04
STC8	0.17	0.15	7.15	4.75	1.78	3.54	0.07	10.46
STC9	0.16	0.15	4.14	7.1	2.02	3.94	0.1	10.51
STC10	0.07	0.1	1.72	1.99	4.33	3.38	0.03	7.29
STC14	0.11	0.13	2.7	3.25	3.14	5.54	0.06	9.38
STC16	0.04	0.03	0.1	0.19	0.11	0.14	4.89	0.59
TO	0.82	0.65	8.88	10.5	7.21	11.27	0.41	39.74
Net	0.36	-0.39	-1.58	0	-0.09	1.89	-0.18	TCI = 5.68
NPDC	5	0	2	3	4	5	2	

**Note**: Results are based on TVP-VAR method with lag length 2 (AIC), 20-step-ahead generalized forecast error variance decomposition and a window size of 200.

The analysis of static spillover effects in both time and frequency domains, presented in Table 4, reveals that overall connectedness is 37.18%, showing that there is indeed spillover connectivity across energy, commodities and spot time charter freight. The average directional spillover connectedness reveals that the largest spillover shocks originates/receive by the spot time charter freight. This implies that time charter freights are strongly integrated within the system, whereas the revised backhaul (STC16), energy (BRENT), and commodities (IRON) market exhibit lesser degrees of interconnectedness. Empirical findings reveal that bulk transportation along the China-Brazil (STC14) route is the primary source of volatility shocks transmitted to other variables (74.80%) and the primary recipient of volatility shocks from other variables (60.57%) in the system at the same time. The results show that the freight market along the China-Brazil route significantly impacts volatility across all variables. Conversely, the future price of iron ore (6.96%) experiences more shocks than transmission shocks, primarily because of the intense volatility of crude oil futures prices. In recent years, there has been substantial growth in free trade within the transportation sector between China and Brazil (STC14). As a result, the transportation market between China and Japan (STC10) has had the most substantial spillover effect, amounting to 29.14%.

In comparison, the contribution of the transportation markets between the Continent and the Mediterranean via China and Japan (STC9) and Gibraltar and Hamburg (STC8) is merely 13.91% and 11.41%, respectively. At the same time, the energy (BRENT) and commodities (IRON) market has a minor spillover connection with time charter freight, contributing 8.53% and 5.88% to others and 7.65% and 12.84% from other components, somewhat each market independently influenced. Interestingly, the energy market can diversify net positive volatility (0.88%) rather than receiving it from other causes. The energy market significantly influences the network due to differences in objectives, preferences, and institutional constraints among economic participants in the spot voyage/time charter freight, energy, and commodity markets. Market connectivity varies across different frequencies, as demonstrated in studies by Chen et al. [54] and Jiang et al. [28]. By employing the TVP-VAR-BK approach, we dissect the total volatility spillovers and examine the transitional links among spot voyages, time charters, energy, and commodity networks. According to Table 4, the total spillover connections are divided into short- and long-term shocks. Short-term connectedness (31.51%) predominantly drives the total connectedness (37.18%), with a lesser contribution from long-term connectedness (5.68%). This indicates that short-term spillover effects are more significant, with shocks to one variable quickly affecting others over a 1 to 28-day period, whereas their impact diminishes over the long term. It is essential to emphasize short-term interconnectedness, as it delineates the adjustment phase for interactions between the shipping freight and commodities markets [54].

Notably, across all frequency bands, the net spillover value for the time charter freight market between China and Brazil (STC14) remains consistently positive (12.34% and 1.89%). This indicates that its overall impact on the system is consistently more significant than that of other indicators, regardless of whether it is in the short or long term. In contrast, the transportation freight market between the Continent and the Mediterranean area (STC9) has a significant impact only in the near term (3.24%) when functioning as a net transmitter of spillovers. However, it does not exert a noteworthy influence in the long run. Intriguingly, the charter freight routes via Gibraltar-Hamburg (STC8) and China-Japan (STC10) are susceptible to changes in other markets in the short term, contributing 3.18% and 4.43%, respectively. However, they have fewer spillovers over the long run. Another important indicator is the commodity price market (IRON), which consistently acts as a transmitter of volatility over both short-term (0.53%) and long-term (0.36%) periods. This finding contrasts with the conclusions of Riaz et al. [14], who demonstrated that the crude oil market remains a net transmitter of volatility within the dry bulk-crude oil relationship and does not exhibit spillover interactions with the Capesize freight (BCI) market. Their study estimated the TCI using only a time-domain TVP-VAR model with an average spot freight index for the various dry bulk freight sub-sectors.

In analyzing the overall spillover connectivity across the spot voyage, energy, and commodity freight networks (Table 5), it is evident that the single voyage freight from Brazil to China (SVC3) significantly contributes to volatility (15.17%) in the interconnected network. This indicates that the SVC3 freight market contributes significantly more volatility (81.85%) than it receives (66.69%) within the system. On the other hand, the commodity price (IRON) is found to be the primary recipient of volatility (9.29%) in the network. The connectivity among the spot voyage, energy, and commodity networks reveals that the crude oil and iron-ore futures markets are primarily influenced by their past dynamics rather than changes in trip freight rates. Given the potential variations in spillover interdependence across different frequency domains due to diverse objectives, preferences, and institutional constraints, it is crucial to elucidate the interrelationships within the network across various frequency bands.

**Table 5:** Time-frequency average spillover connectedness along spot voyage route.

	BRENT	IRON	SVC2	SVC3	SVC5	SVC7	SVC17	FROM
Total spillover connectedness based on TVP-VAR-DY								
BRENT	87.75	2.45	2.06	1.46	1.97	2.39	1.91	12.25
IRON	4.91	82.81	2.48	2.71	2.28	2.56	2.27	17.19
SVC2	1.25	1.18	30.8	19.37	12.23	19.68	15.48	69.2
SVC3	1.03	1.07	16.02	33.31	14.41	14.52	19.63	66.69
SVC5	0.96	1.1	13.72	17.64	39.46	12.31	14.8	60.54
SVC7	1.26	1.1	19.5	17.68	11.01	35.7	13.74	64.3
SVC17	1.22	1.01	14.28	22.99	13.88	13.18	33.43	66.57
TO	10.64	7.9	68.06	81.85	55.8	64.65	67.83	356.74
Net	-1.61	-9.29	-1.14	15.17	-4.75	0.35	1.26	TCI = 50.96
NPDC	1	0	3	6	2	4	5	
Short-term (1–28 Days) spillover connectedness based on TVP-VAR-DY-BK								
BRENT	83.48	2.3	1.87	1.37	1.79	2.26	1.77	11.36
IRON	4.18	77.07	2.23	2.48	2.06	2.3	2.07	15.32
SVC2	0.93	0.9	23.94	14.2	9.48	14.57	11.06	51.14
SVC3	0.77	0.86	13.03	28.01	12.14	11.75	16.05	54.59
SVC5	0.78	0.96	11.98	15.5	36.18	10.6	12.91	52.72
SVC7	0.97	0.87	15.01	13.16	8.78	28.65	10.11	48.91
SVC17	0.83	0.78	11.18	18.44	11.34	10.23	28.18	52.81
TO	8.48	6.68	55.3	65.15	45.59	51.7	53.97	286.86
Net	-2.88	-8.64	4.16	10.56	-7.14	2.78	1.16	TCI = 40.98
NPDC	1	0	5	6	2	4	3	
Long-term (28 Days and above) spillover connectedness based on TVP-VAR-DY-BK								
BRENT	4.28	0.14	0.19	0.09	0.19	0.13	0.14	0.89
IRON	0.72	5.73	0.25	0.22	0.23	0.26	0.2	1.87
SVC2	0.32	0.28	6.86	5.17	2.75	5.12	4.42	18.06
SVC3	0.26	0.21	2.99	5.31	2.27	2.78	3.59	12.1
SVC5	0.18	0.14	1.74	2.15	3.28	1.71	1.9	7.82
SVC7	0.29	0.22	4.49	4.52	2.23	7.05	3.63	15.38
SVC17	0.39	0.23	3.1	4.55	2.54	2.95	5.24	13.76
TO	2.16	1.23	12.76	16.71	10.21	12.95	13.87	69.88
Net	1.27	-0.65	-5.3	4.61	2.39	-2.43	0.11	TCI = 9.98
NPDC	5	2	0	4	6	2	2	

**Note**: Results are based on TVP-VAR method with lag length 3 (AIC), 20-step-ahead generalized forecast error variance decomposition and a window size of 200.

There is a strong interrelationship between energy and commodities markets in spot voyage and time charter networks. The crude oil (BRENT) market transmits 4.58% volatility to the iron-ore (IRON) market and receives a 2.16% spillover from iron-ore. Similar patterns are observed in the voyage freight network, where the crude oil price transfers 4.91% overflow to iron-ore and receives 2.45% simultaneously. These findings align with the conclusions drawn by Chen et al. [9], who identified a time-varying interrelationship among dry bulk freight (BDI), crude oil, and the iron-ore market using the BEKK-GARCH-X approach. They also revealed lead-lag relationships using each as an exogenous variable for different sub-samples. However, their findings did not provide the direction of spillover interconnectedness and time-domain volatility spillover. Additionally, they used average spot freight of dry bulk, which may not capture the individual characteristics of the transportation market in various routes for the spot voyage and time charter freight.

The results from the frequency domain spillover analysis indicate an asymmetric spillover connectedness across various frequency domains. The spillover responses of all indicators demonstrate a greater sensitivity within the system during short-term periods, reaching 40.98%, while long-term shocks contribute a lower proportion, at 9.98%. Specifically, the returns of the iron ore commodity price (IRON) show heightened sensitivity in the short term, declining by 8.64% due to influences from other freight markets. This is followed closely by the Australia-Qingdao freight market (SVC5), which records a response of approximately -7.14%. In contrast, the voyage route along the Tubarao-Qingdao freight market (SVC3) serves as a primary transmitter, channeling 10.56% of spillovers into the system, a significantly higher contribution compared to other routes, including SVC2, SVC7, and SVC17.

Additionally, in the long term, the energy market (BRENT) shifts its role from being a net receiver to a net transmitter of spillovers. While IRON consistently functions as a spillover receiver, SVC3 and SVC17 act as steady transmitters across both short and long-term periods. Interestingly, the spillover direction for the voyage routes SVC2 and SVC7 transitions from positive to negative between these periods. Meanwhile, SVC5, which initially absorbs spillovers at -7.14%, ultimately transmits a moderate level of volatility, amounting to 2.39%, in the long-term periods.

### 5.2. Dynamic spillover connectedness along time charter routes

**Fig 2:**
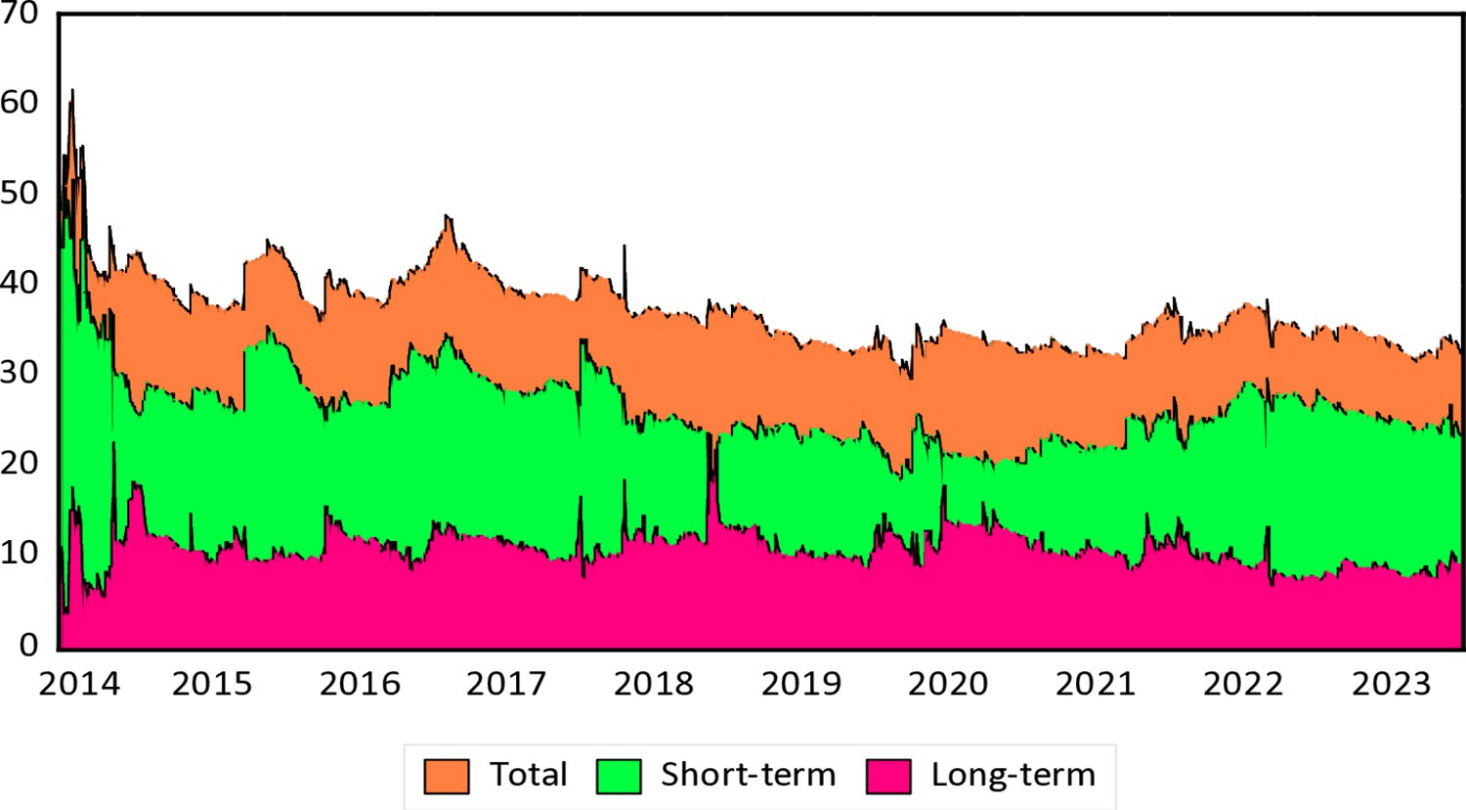
Dynamic time-frequency overall spillover connectedness within spot time charter market. Note: Horizontal axis represents the time dimension and vertical axis represents the intensity of the spillover effect (%). Results are based on TVP-VAR method with lag length 2 (AIC), 20-step-ahead generalized forecast error variance decomposition and a window size of 200.

The analysis in this section focuses on the examination of total and net directional time-varying volatility spillovers between spot time charter freight, energy, and commodity markets. The vertical axis represents the intensity of the spillover effect in terms of net pairwise directional connectedness. Higher values indicate stronger spillover effects from one variable to another. The horizontal axis indicates the time dimension, showing how the spillover effects evolve over time. This allows observation of how the strength and direction of these connections change across different time periods, particularly under various economic or geopolitical conditions. The aim is to understand how risk spillovers have evolved, especially during significant events like the oil crisis in 2016, the US-China trade war in 2018, the COVID-19 pandemic, and the Russia-Ukraine conflict in 2022. This analysis covers both short- and long-term investment horizons and utilizes a TVP-VAR model that considers both time and frequency domains to estimate dynamic spillover connections. The study involves the use of the TVP-VAR-DY model to examine dynamic patterns over time and the TVP-VAR-BK model to analyze spillover interactions within economic variables across different frequencies. It employs a 20-step forecast and a 200-day rolling window, with optimal lag lengths determined using the Akaike Information Criterion (AIC). The results of the dynamic Total Connectedness Index (TCI) across the spot time charter, energy, and commodities networks over short and long-term periods are presented in Fig 2.

**Fig 3:**
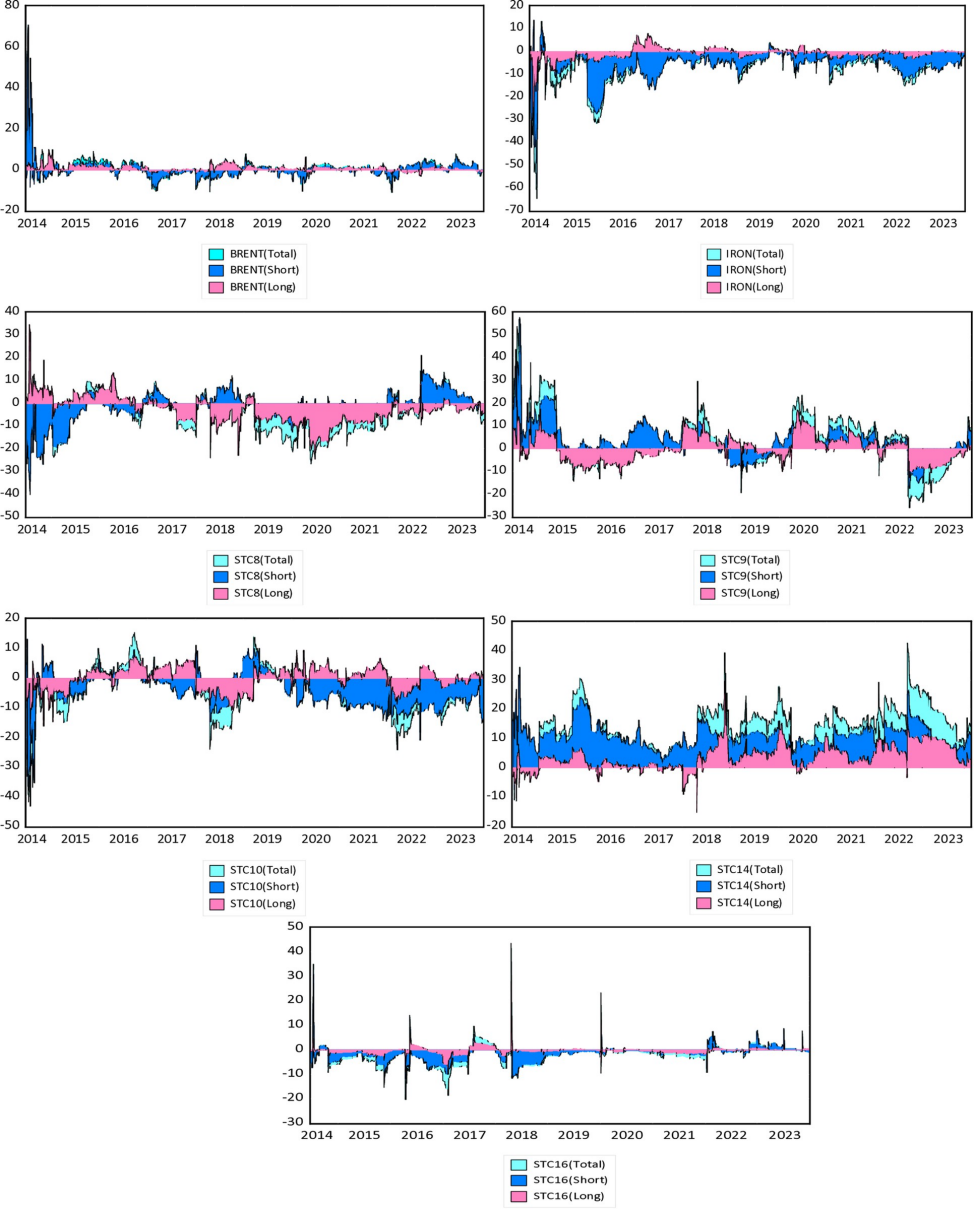
Dynamic time-frequency net spillover connectedness within spot time charter market (Net). Note: Horizontal axis represents the time dimension and vertical axis represents the intensity of the spillover effect (%). Results are based on TVP-VAR method with lag length 2 (AIC), 20-step-ahead generalized forecast error variance decomposition and a window size of 200.

Fig 3 illustrates the net directional spillovers. There is evidence of dynamic spillover interactions across various investment horizons, including total, short-term, and long-term. The average TCI (Fig 2) shows intermittent significant increases and decreases when analyzing dynamic spillover connections across different frequency bands. The dynamic TCI typically ranges between 35% and 65%. During extreme market conditions, such as the oil crisis (44%), the US-China trade war (47%), COVID-19 (35%), and the Russia-Ukraine conflict (38%), general volatility spillovers increase dramatically. The TCI volatility among time charter, energy, and commodity markets notably spiked during the short term of the US-China trade war compared to other economic and financial shocks. However, this pattern is not observed in the long term. This suggests that the impacts of tariffs and trade tensions between the US and China have a more noticeable effect on short-term risk spillovers than financial and geopolitical shocks. Conversely, the long-term effects of geopolitical tensions resulting from the war are more significant than those of the pandemic. This conclusion aligns with the findings of Lin et al. (2023), who noted substantial fluctuations in dynamic TCI due to geopolitical shocks within the BDI, energy, and petrochemical commodities network.

The average dynamic TCI can be divided into two distinct frequency bands, similar to the static data. Short-term connections are the strongest, followed by long-term connections, indicating that our research demonstrates that short-term variables primarily influence interconnectivity. The results of the Dynamic TCI analysis underscore the market’s response to significant global disruptions over short and long run spillovers. The increase in short-term connectivity (red) during the 2016 financial crisis, driven by events such as the Chinese stock market decline, reflects rapid and widespread reactions as markets swiftly conveyed uncertainty. The long-term impacts (green) were limited, indicating that markets swiftly adapted post-crisis. In 2018, the US-China trade dispute triggered another increase in short-term connectivity, highlighting the swift impact of tariffs and trade tensions on global markets. The analysis indicates that while the trade war caused short-term disruptions, it did not significantly alter long-term market ties. The COVID-19 pandemic in 2020 resulted in an unprecedented surge in short-term connections due to the global economic shutdown, leading to exceptional market volatility. However, long-term connectivity was less affected, indicating quick but temporary changes. Similarly, the Russia-Ukraine crisis 2022 resulted in limited short-term spillovers, reflecting rapid responses to geopolitical concerns. Nonetheless, long-term connectivity remained subdued, suggesting that the markets were resilient in absorbing these shocks without prolonged disturbances.

Next, we will analyze the dynamic net spillover within the time charter-energy-commodity network across various frequencies. Fig 3 visually represents the dynamic net spillover, which aligns with the average net spillover results outlined in Table 4. A positive value indicates that the variable can transmits volatility than receiving shocks into the system. Reciprocally, a negative value in net spillover implies that it is sensitive to the fluctuations of other variables functions in the network. Building on our previous examination of average net spillover, we notice that the dynamic net spillover graphs reveal fluctuations in each variable’s net spillover over time, signifying an increase or decrease based on whether the variable serves as a shock absorber or a transmitter.

A closer review of the graphs reveals that the intensity of shock transmission or reception notably amplifies during crises for most variables over the short-term. The net spillover effects experienced by various time charter markets underscore significant disparities in their responses to short-term (red) and long-term (green) shocks. Notably, Market STC14 consistently records the highest short-term positive spillovers, exceeding 40%, particularly during significant events such as the 2016 oil crisis, the US-China trade war in 2018, the COVID-19 pandemic in 2020, and the Russia-Ukraine conflict in 2022. In contrast, the BRENT, IRON, and Spot time C16 markets, influenced by global commodity prices, exhibit substantial short-term spillovers of around 40% during these international events, indicating a heightened sensitivity to rapid shocks compared to other markets. Across all markets, long-term spillovers remain relatively low at around 10%, even during major geopolitical events such as the Russia-Ukraine crisis in 2022. This suggests that while these markets respond significantly to short-term shocks, the long-term impacts are limited, stabilizing prices following initial disruptions.

The overall net spillover effect of the crude oil market (BRENT) on the system consistently remained low over the entire period. The unusual market conditions impacted the net spillover dynamics in 2016, 2018, 2020, and 2022. The price volatility of crude oil has a dual impact on the demand for maritime transportation of crude oil and the transportation expenditures of vessel carriers (Shi et al., 2013), consequently directly influencing the freight rate. IRON demonstrates that this series continually experiences short-term net shocks, which also holds for long-term dynamics. The analysis of STC14 time charter freight indicates that this series consistently transmits shocks in the short-term, and this pattern persists for long-term dynamics as well. The data suggests that STC8 typically experiences volatility as a net acceptor after 2016. Despite STC8 undergoing significant spillover before and during COVID-19, this market transformed into a net positive spillover entity during the Russian-Ukrainian conflict in 2022. STC9 and STC14, primarily functioning as net transmitters, have a significant positive spillover effect on other submarkets, particularly in 2016, 2018, 2020, and 2022. This is attributed to the oil crisis, the trade war between China and the US, the impact of Covid-19, and the conflicts between Russia and Ukraine.

It is worth noting that the Russian-Ukrainian conflict has a more enduring effect on STC14, while the trade difficulties between the US and China negatively impact the STC9 freight market. These dynamics result from the escalating Russian-Ukrainian conflict and the ongoing trade war, leading to trade and shipping market limitations. The significance of STC10 in the system grows after 2018, where it shifts to receiving volatility, particularly during the downturns caused by the COVID-19 and the Russia-Ukraine war in 2020 and 2022. Notably, STC10 functions as a net emitter during the 2016 oil crisis and the US-China trade war in 2018. Likewise, Fig 3 shows that key events also significantly impact the STC16 freight market. The 2018 US-China trade war leads to increased upper-tailed spillovers in STC16, and other significant events (oil crisis, COVID-19 pandemic, Russia-Ukraine conflict) result in net positive spillover shocks for this route. Under normal market conditions, the net spillover effects show negative spillovers. Throughout the sample, the short-term shocks is more pronounced than long-term spillover in the system.

### 5.3. Dynamic spillover connectedness along voyage freight routes

**Fig 4:**
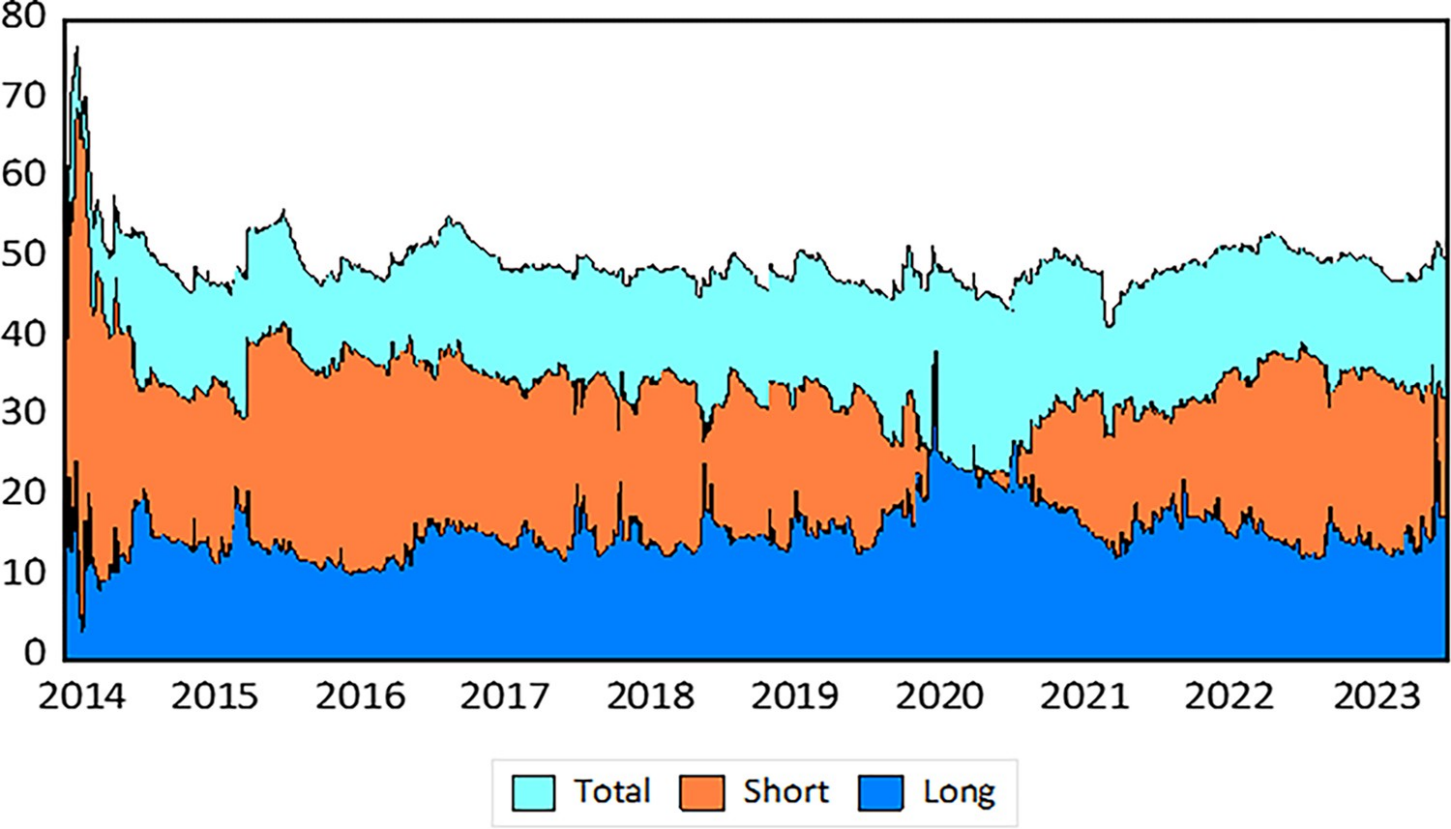
Dynamic time-frequency overall spillover connectedness within spot voyage market (Net). Note: Horizontal axis represents the time dimension and vertical axis represents the intensity of the spillover effect (%). Results are based on TVP-VAR method with lag length 2 (AIC), 20-step-ahead generalized forecast error variance decomposition and a window size of 200.

The following section focuses on analyzing the total and net directional time-varying volatility spillovers between spot voyage charter freight, energy, and commodity markets. Fig 4 reveals the dynamic total spillover. The analysis of dynamic TCI and net spillover effects among the spot voyage, energy, and commodity markets reveals that spillover connections vary, unlike those in the time charter freight market. It is noteworthy that over 50% of the forecast errors stem from spillovers between markets, underscoring their high vulnerability to significant shocks such as the 2016 oil crisis, the 2018 US-China trade war, the COVID-19 pandemic in 2020, and the Russia-Ukraine conflict in 2022. Fig 4 presents the dynamic time-frequency spillover relationship among spot voyage freight, energy, and commodity markets. The following section focuses on analyzing the total and net directional time-varying volatility spillovers between spot voyage charter freight, energy, and commodity markets.

**Fig 5:**
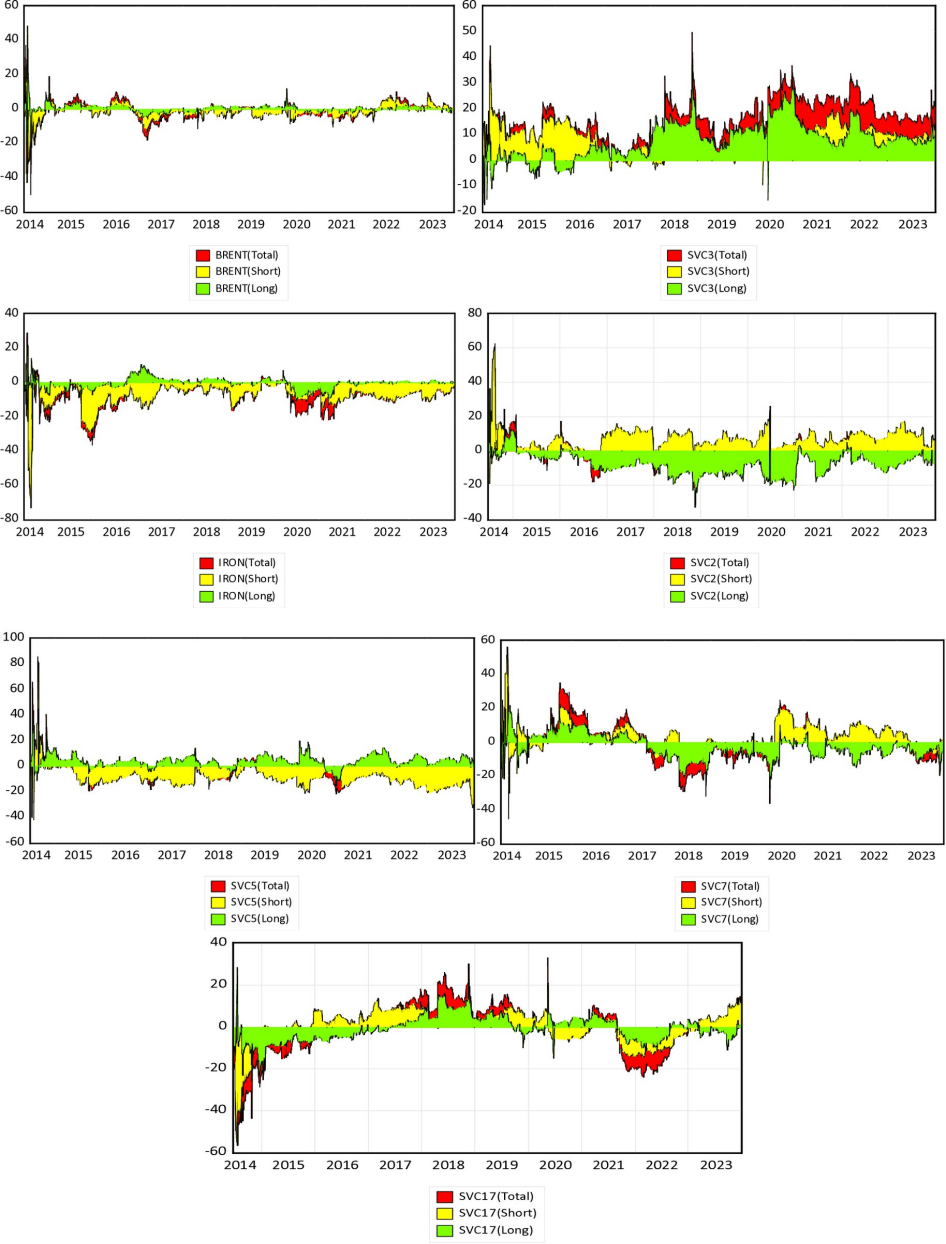
Dynamic time-frequency net spillover connectedness within spot voyage market (Net). Note: Horizontal axis represents the time dimension and vertical axis represents the intensity of the spillover effect (%). Results are based on TVP-VAR method with lag length 3 (AIC), 20-step-ahead generalized forecast error variance decomposition and a window size of 200.

Fig 4 demonstrates the dynamic Total Connectedness Index (TCI) at different frequencies for both short- and long-term periods, as well as the total spillover connectedness. Fig 5 illustrates the net directional spillover. An analysis of the dynamic TCI and net spillover effects among the spot voyage, energy, and commodity markets shows that spillover connections vary, unlike those in the time charter freight market. It is important to note that over 50% of the forecast errors are due to spillovers between markets, highlighting their high vulnerability to significant shocks such as the 2016 oil crisis, the 2018 US-China trade war, the COVID-19 pandemic in 2020, and the Russia-Ukraine conflict in 2022. Fig 4 presents the dynamic time-frequency spillover relationship among spot voyage freight, energy, and commodity markets. The average TCI plot shaded in black reveals a heightened degree of spillover connection over the first period from 2015 to 2016, reaching about 80%, which aligns with the 2016 oil crisis. This peak shows robust connectivity across many markets, primarily driven by short-term spillovers (red area), implying fast market changes. Following this peak, there is a continuous reduction and stability of spillover linkages between 2016 and 2019, with values ranging from 40% to 60%, suggesting that the markets responded to earlier shocks while maintaining a balance between short-term and long-term repercussions.

During important events like the 2018 US-China trade war and the 2020 COVID-19 pandemic, there are substantial fluctuations in connection, implying heightened volatility and dynamic interactions across the markets, driven again by short-term spillovers. The spillover link increased once again in 2020, surpassing 60%, highlighting the global market’s vulnerability to the economic consequences of the COVID-19 pandemic. This elevated level persists through 2022, underscoring the lasting impact of the pandemic and the Russia-Ukraine conflict, with short-term spillovers remaining strong, indicating rapid market reactions to future shocks. Toward the latter part of the period, there is a noticeable surge in total spillover connections, indicating increased volatility and heightened interconnectedness due to current geopolitical tensions and economic concerns. The consistent prevalence of short-term spillovers during these periods underscores the markets’ immediate response to acute shocks. Meanwhile, the notably lower frequency of long-term spillovers (green zone) suggests fewer lasting changes.

The IRON pricing shows a consistent negative spillover impact in both short- and long-term dynamics. During 2015–2016 and 2020, the net spillover effects of IRON suggest increased sensitivity within the network, influenced significantly by global oil prices (45%) and the COVID-19 pandemic (40%). Long-term crude oil shocks had a more significant impact than short-term shocks during the 2016 oil price crisis. Net spillover effects turned negative during the 2018 US-China trade war and the 2022 Russia-Ukraine conflict, though BRENT continued to transmit shocks to the system during COVID-19 in 2020. The net spillover of SVC2 indicates it primarily transmits in the short term but receives spillovers in the long term. Contrastingly, SVC3 demonstrates strong spillover transmission over the whole frequency. The consequences of the 2018 US-China trade conflict signified a shift from short-run to long-run transmission.

However, during the Russia-Ukraine geopolitical tensions, SVC3 predominantly addressed short-term shocks. Likewise, SVC5 exhibits unfavorable overall spillover volatility in the short term but minimal long-term impact, restricting its transmission of volatility to the system. The overall spillover effect is especially evident during significant events in 2016, 2018, 2020, and 2022. From 2015 to 2016, SVC7 served as a significant conveyor of volatility in the short term. Nevertheless, during the 2018 US-China trade conflict and the period of COVID-19 in 2020, SVC7 began to experience volatility in overall frequency domain. The SVC7 market transmits spillovers in short-term dynamics but receives spillovers in the post-COVID era (2021–2024). Conversely, SVC17 encountered disturbances in both short- and long-term dynamics during the 2016 oil crisis but generally acted as a buoyant net transmitter of disturbances at other times.

### 5.4. Robustness analysis

The prediction horizon has been set to 10, with a rolling window length of 250 units, while other indications have been retained to ensure the model’s robustness. The results shown in Table 6 (time charter route) and Table 7 (voyage freight route) demonstrate that the overall, short-term, and long-term spillovers closely align with the findings from both the static and dynamic spillover analyses. It is interesting that short-term dynamics have the largest spillover intensity, followed by long-term shocks in both the time charter and route charter networks. For instance, along the trip charter freight routes, the Total Connectedness Index (TCI) at a 10-step prediction horizon is 50.89 for overall, 33.21 for short-term, and 17.68 for long-term, whereas with a rolling window size of 250 units, these values are 50.99, 41.12, and 9.87, respectively. In the time charter freight routes, the overall, short-term, and long-term TCI values for a 10-step forecast horizon are 37.18, 26.42, and for a 250-unit window size, they are 37.17, 31.51, and 5.66, respectively. Furthermore, in both channels, long-term spillover connection demonstrates a continuous tendency in which crude oil (BRENT) functions as a transmitter, while iron ore (IRON) operates as a receiver. Additionally, in the voyage freight route, we identify its role as both a short-term and long-term transmitter of spillovers, a conclusion confirmed by the data in Table 5.

**Table 6:** Robustness for spot time charter market.

	RCRUDE	RIRON	STC14	STC9	STC10	STC14	STC16	FROM
Total spillover connectedness based on TVP-VAR-DY								
RCRUDE	(92.36,92.37)	(2.16,2.07)	(1.18,1.2)	(1.25,1.27)	(1,0.99)	(1.12,1.09)	(0.95,1.02)	(7.64,7.63)
RIRON	(4.58,4.68)	(87.16,87.19)	(1.63,1.64)	(2.03,1.92)	(1.6,1.59)	(2.22,2.18)	(0.79,0.8)	(12.84,12.81)
STC14	(1.03,1.05)	(0.97,0.98)	(43.13,43.16)	(23.95,23.9)	(11.25,11.24)	(19.16,19.17)	(0.5,0.5)	(56.87,56.84)
STC9	(0.93,0.91)	(0.81,0.8)	(21.55,21.67)	(40.88,40.87)	(13.02,13.01)	(21.87,21.79)	(0.94,0.95)	(59.12,59.13)
STC10	(0.57,0.6)	(0.74,0.74)	(11.41,11.51)	(13.91,13.87)	(43.45,43.39)	(29.14,29.12)	(0.78,0.79)	(56.55,56.61)
STC14	(0.65,0.66)	(0.77,0.77)	(15.36,15.48)	(19.14,19.01)	(24.05,24.04)	(39.43,39.42)	(0.6,0.61)	(60.57,60.58)
STC16	(0.75,0.69)	(0.41,0.42)	(0.99,0.99)	(2.1,2.11)	(1.11,1.11)	(1.29,1.3)	(93.34,93.38)	(6.66,6.62)
TO	(8.51,8.59)	(5.86,5.77)	(52.11,52.49)	(62.36,62.09)	(52.04,51.97)	(74.8,74.65)	(4.56,4.67)	(260.25,260.22)
Net	(0.87,0.96)	(-6.97,-7.04)	(-4.75,-4.35)	(3.24,2.96)	(-4.51,-4.64)	(14.23,14.07)	(-2.1,-1.95)	TCI = (37.18,37.17)
NPDC	(1,1)	(0,0)	(4,4)	(5,5)	(3,3)	(6,6)	(2,2)	
Short-term (1–28 Days) spillover connectedness based on TVP-VAR-BK								
RCRUDE	(84.3,88.15)	(1.98,1.98)	(1.07,1.14)	(1.05,1.17)	(0.9,0.94)	(0.95,1)	(0.81,0.94)	(6.76,7.16)
RIRON	(4.03,4.39)	(75.87,81.25)	(1.31,1.47)	(1.6,1.73)	(1.4,1.48)	(1.86,2)	(0.64,0.72)	(10.85,11.79)
STC14	(0.71,0.87)	(0.69,0.83)	(29.59,36.01)	(15,19.16)	(7.87,9.46)	(12.46,15.64)	(0.37,0.43)	(37.1,46.39)
STC9	(0.63,0.75)	(0.53,0.64)	(13.73,17.53)	(27.47,33.81)	(9.18,10.99)	(14.41,17.89)	(0.75,0.85)	(39.23,48.66)
STC10	(0.44,0.52)	(0.55,0.64)	(8.14,9.78)	(10.14,11.88)	(35.21,39.07)	(22.72,25.75)	(0.72,0.76)	(42.71,49.33)
STC14	(0.45,0.55)	(0.52,0.64)	(10.24,12.77)	(12.99,15.8)	(18.08,20.91)	(28.9,33.9)	(0.5,0.55)	(42.78,51.22)
STC16	(0.68,0.65)	(0.36,0.39)	(0.8,0.89)	(1.75,1.92)	(0.91,1.01)	(1.03,1.16)	(84.02,88.48)	(5.54,6.03)
TO	(6.94,7.75)	(4.63,5.12)	(35.29,43.59)	(42.53,51.66)	(38.35,44.79)	(53.43,63.44)	(3.79,4.24)	(184.97,220.57)
Net	(0.19,0.59)	(-6.22,-6.68)	(-1.81,-2.8)	(3.3,3)	(-4.36,-4.54)	(10.66,12.22)	(-1.75,-1.79)	TCI = (26.42,31.51)
NPDC	(1,1)	(0,0)	(4,4)	(5,5)	(3,3)	(6,6)	(2,2)	
Long-term (28 Days and above) spillover connectedness based on TVP-VAR-BK								
RCRUDE	(8.06,4.21)	(0.18,0.09)	(0.11,0.06)	(0.19,0.11)	(0.1,0.05)	(0.17,0.09)	(0.13,0.08)	(0.88,0.48)
RIRON	(0.55,0.29)	(11.29,5.94)	(0.32,0.17)	(0.42,0.2)	(0.2,0.1)	(0.35,0.18)	(0.14,0.08)	(1.98,1.02)
STC14	(0.32,0.18)	(0.28,0.15)	(13.54,7.15)	(8.95,4.74)	(3.38,1.78)	(6.7,3.53)	(0.13,0.07)	(19.76,10.45)
STC9	(0.29,0.16)	(0.28,0.15)	(7.82,4.13)	(13.41,7.06)	(3.84,2.01)	(7.46,3.9)	(0.19,0.1)	(19.89,10.47)
STC10	(0.13,0.07)	(0.19,0.1)	(3.27,1.73)	(3.77,1.98)	(8.24,4.32)	(6.43,3.37)	(0.06,0.03)	(13.84,7.29)
STC14	(0.21,0.11)	(0.25,0.13)	(5.12,2.71)	(6.14,3.21)	(5.97,3.13)	(10.52,5.52)	(0.11,0.06)	(17.8,9.36)
STC16	(0.07,0.03)	(0.05,0.03)	(0.18,0.1)	(0.36,0.19)	(0.2,0.11)	(0.26,0.14)	(9.32,4.9)	(1.12,0.59)
TO	(1.56,0.84)	(1.23,0.66)	(16.82,8.9)	(19.83,10.43)	(13.69,7.18)	(21.37,11.21)	(0.77,0.43)	(75.28,39.65)
Net	(0.68,0.37)	(-0.75,-0.36)	(-2.94,-1.56)	(-0.06,-0.04)	(-0.15,-0.1)	(3.58,1.85)	(-0.35,-0.16)	TCI = (10.75,5.66)
NPDC	(5,5)	(0,0)	(2,2)	(3,3)	(4,4)	(5,5)	(2,2)	

**Note**: Results are based on time and frequency domain TVP-VAR method with lag length 2 (AIC). Values in parenthesis (), first one represents 10 step ahead forecast variance decomposition and 2^nd^ one represents for window size 250.

**Table 7:** Robustness for spot voyage freight market.

	RCRUDE	RIRON	SVC2	SVC3	SVC5	SVC7	SVC17	FROM
Total spillover connectedness based on TVP-VAR-DY								
RCRUDE	(87.82,87.59)	(2.44,2.42)	(2.06,2.14)	(1.45,1.5)	(1.95,1.9)	(2.39,2.55)	(1.89,1.9)	(12.18,12.41)
RIRON	(4.85,4.83)	(82.95,82.41)	(2.46,2.55)	(2.7,2.88)	(2.25,2.27)	(2.53,2.79)	(2.26,2.26)	(17.05,17.59)
SVC2	(1.19,1.17)	(1.16,1.32)	(30.86,30.86)	(19.39,19.33)	(12.23,12.16)	(19.7,19.72)	(15.47,15.44)	(69.14,69.14)
SVC3	(0.98,0.97)	(1.04,1.16)	(16.03,16.08)	(33.37,33.38)	(14.41,14.27)	(14.53,14.45)	(19.64,19.7)	(66.63,66.62)
SVC5	(0.92,0.93)	(1.08,1.18)	(13.73,13.75)	(17.66,17.57)	(39.5,39.5)	(12.31,12.24)	(14.8,14.84)	(60.5,60.5)
SVC7	(1.24,1.21)	(1.07,1.19)	(19.5,19.61)	(17.69,17.64)	(11.01,10.86)	(35.75,35.86)	(13.73,13.63)	(64.25,64.14)
SVC17	(1.12,1.05)	(0.99,1.01)	(14.3,14.44)	(23.02,23.1)	(13.87,13.72)	(13.2,13.19)	(33.49,33.48)	(66.51,66.52)
TO	(10.3,10.16)	(7.78,8.28)	(68.07,68.58)	(81.91,82.02)	(55.74,55.17)	(64.67,64.94)	(67.8,67.78)	(356.26,356.93)
Net	(-1.88,-2.25)	(-9.26,-9.31)	(-1.07,-0.56)	(15.28,15.4)	(-4.77,-5.33)	(0.42,0.8)	(1.28,1.26)	TCI = (50.89,50.99)
NPDC	(1,1)	(0,0)	(3,3)	(6,6)	(2,2)	(4,4)	(5,5)	
Short-term (1–28 Days) spillover connectedness based on TVP-VAR-BK								
RCRUDE	(79.77,83.41)	(2.19,2.27)	(1.73,1.94)	(1.3,1.4)	(1.62,1.75)	(2.16,2.41)	(1.66,1.76)	(10.65,11.52)
RIRON	(3.78,4.29)	(72.01,76.69)	(2.05,2.27)	(2.3,2.62)	(1.9,2.08)	(2.1,2.47)	(1.94,2.08)	(14.07,15.82)
SVC2	(0.72,0.89)	(0.68,1.03)	(18.57,23.94)	(10.2,14.14)	(7.42,9.47)	(10.64,14.58)	(7.68,11.01)	(37.33,51.14)
SVC3	(0.61,0.77)	(0.68,0.96)	(10.66,13.06)	(23.65,28.07)	(10.35,12.1)	(9.58,11.68)	(13.15,16.11)	(45.03,54.69)
SVC5	(0.68,0.81)	(0.83,1.04)	(10.59,11.97)	(13.77,15.41)	(33.46,36.31)	(9.23,10.5)	(11.37,12.9)	(46.46,52.62)
SVC7	(0.76,0.97)	(0.67,0.98)	(11.52,15.08)	(9.67,13.13)	(7.05,8.73)	(23.05,28.77)	(7.35,10.02)	(37.02,48.92)
SVC17	(0.66,0.82)	(0.61,0.83)	(8.72,11.3)	(14.69,18.55)	(9.35,11.34)	(7.9,10.25)	(23.89,28.28)	(41.94,53.1)
TO	(7.21,8.57)	(5.66,7.11)	(45.27,55.62)	(51.93,65.25)	(37.68,45.49)	(41.61,51.89)	(43.14,53.89)	(232.5,287.82)
Net	(-3.43,-2.96)	(-8.41,-8.71)	(7.93,4.49)	(6.89,10.56)	(-8.77,-7.14)	(4.59,2.97)	(1.19,0.79)	TCI = (33.21,41.12)
NPDC	(1,1)	(0,0)	(6,5)	(5,6)	(2,2)	(4,4)	(3,3)	
Long-term (28 Days and above) spillover connectedness based on TVP-VAR-BK								
RCRUDE	(8.05,4.18)	(0.25,0.15)	(0.33,0.2)	(0.16,0.1)	(0.33,0.15)	(0.23,0.14)	(0.23,0.14)	(1.53,0.89)
RIRON	(1.07,0.54)	(10.94,5.72)	(0.41,0.29)	(0.39,0.26)	(0.35,0.18)	(0.44,0.32)	(0.32,0.18)	(2.98,1.77)
SVC2	(0.47,0.28)	(0.48,0.28)	(12.29,6.92)	(9.18,5.19)	(4.82,2.68)	(9.05,5.14)	(7.8,4.43)	(31.8,18)
SVC3	(0.37,0.19)	(0.36,0.2)	(5.36,3.01)	(9.71,5.31)	(4.06,2.17)	(4.95,2.77)	(6.49,3.59)	(21.6,11.93)
SVC5	(0.25,0.12)	(0.25,0.14)	(3.14,1.78)	(3.89,2.16)	(6.04,3.19)	(3.08,1.74)	(3.43,1.94)	(14.05,7.88)
SVC7	(0.48,0.24)	(0.4,0.22)	(7.97,4.53)	(8.03,4.51)	(3.97,2.13)	(12.7,7.09)	(6.38,3.61)	(27.23,15.22)
SVC17	(0.46,0.23)	(0.38,0.19)	(5.58,3.14)	(8.34,4.55)	(4.52,2.37)	(5.3,2.94)	(9.6,5.2)	(24.57,13.42)
TO	(3.08,1.6)	(2.12,1.17)	(22.8,12.95)	(29.99,16.77)	(18.05,9.68)	(23.05,13.05)	(24.66,13.89)	(123.76,69.11)
Net	(1.55,0.71)	(-0.86,-0.6)	(-9,-5.05)	(8.39,4.84)	(4,1.81)	(-4.17,-2.17)	(0.09,0.47)	TCI = (17.68,9.87)
NPDC	(5,5)	(2,1)	(0,1)	(4,4)	(6,6)	(2,2)	(2,2)	

**Note**: Results are based on time and frequency domain TVP-VAR method with lag length 3 (AIC). Values in parenthesis (), first one represents 10 step ahead forecast variance decomposition and 2^nd^ one represents for window size 250.

## 6. Conclusion and policy implication

This research employed TVP-VAR model and finding reveals a time-varying asymmetric relationship among the charter-energy-commodity network. The crude oil prices consistently transmit short and long terms shocks in the time charter route but act as a positive net transmitter over the long-term periods for voyage route plan. The SVC3 and STC14, consistently transmits spillovers in the short and long-term horizons across the time as well as voyage route plan. In contrast, the Continent-Mediterranean (STC9) transmits shocks in the short term but has no significant long-term effect. The Australia-China route (SVC5) initially absorbs short-term volatility but transitions to transmitting shocks over the long term, while the Tubarao-Rotterdam (SVC2) and Bolivar-Rotterdam (SVC7) routes contribute to short-term volatility and are affected by considerable long-term volatility. The crude oil market (BRENT) absorbs short-term shocks however transmits insignificant volatility in the long-term periods, whereas commodity prices (IRON) experience substantial short- and long-term shocks, with a more pronounced impact in the short term.

While our study offers practical and theoretical contributions, several areas remain open for future research. Future studies could examine how shocks from trade and economic policy uncertainty, as well as investor sentiment, impact return transmission in the freight, energy, and commodities markets.
